# MicroRNA-200c in Cancer Generation, Invasion, and Metastasis

**DOI:** 10.3390/ijms26020710

**Published:** 2025-01-16

**Authors:** Honghao Guo, Ning Zhang, Tao Huang, Na Shen

**Affiliations:** Department of Breast and Thyroid Surgery, Union Hospital, Tongji Medical College, Huazhong University of Science and Technology, 1277 Jiefang Avenue, Wuhan 430022, China; guohonghao0518@163.com (H.G.); zhangning_uh@hust.edu.cn (N.Z.); huangtaowh@hust.edu.cn (T.H.)

**Keywords:** microRNA-200c, tumorigenesis, invasion, metastasis

## Abstract

MicroRNA-200c (miR-200c) is increasingly recognized as a crucial small RNA molecule that plays a significant and multifaceted role in the complex processes of tumor development, invasion, and metastasis across various types of cancers. Recent studies have compellingly demonstrated that miR-200c exerts its influence on tumor biology by meticulously regulating a range of critical processes, including cell proliferation, apoptosis, epithelial–mesenchymal transition (EMT), and cell migration, all of which are essential for the progression and aggressiveness of tumors. This comprehensive review aims to summarize the expression characteristics and functional implications of miR-200c across a diverse array of tumor types, delving into its potential utility as both a biomarker for early detection and a therapeutic target in the realm of cancer treatment. By synthesizing current research findings and insights, we aspire to provide valuable information that could significantly enhance early diagnostic capabilities and inform the strategic development of targeted therapy approaches in oncology.

## 1. Introduction

**Figure 1:**
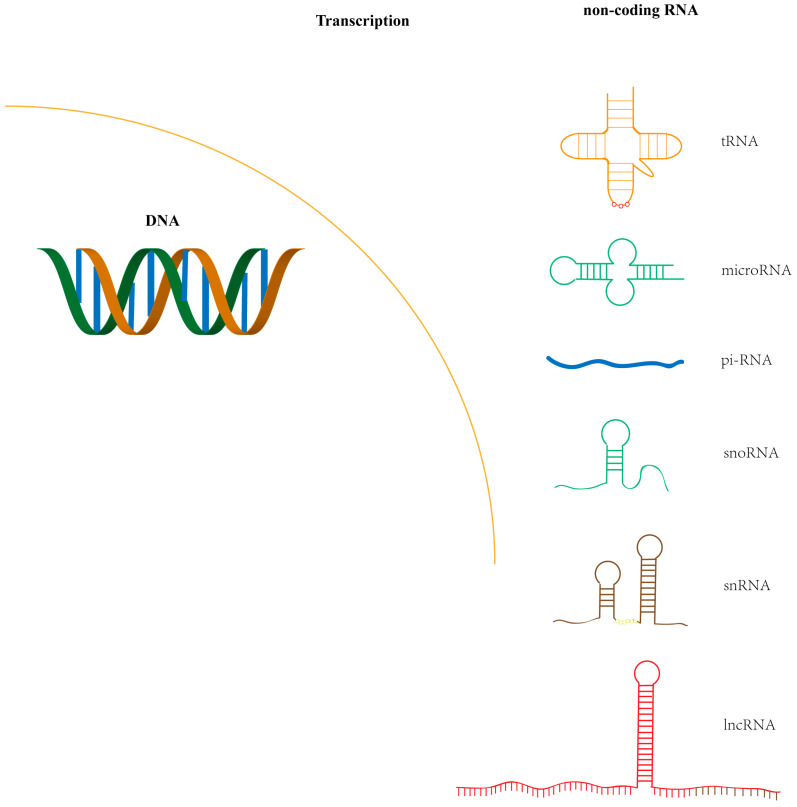
Types and structures of non-coding RNAs.

The most extensively studied genes in the human genome are protein-coding genes, yet these constitute less than 2% of the entire genome [1,2,3]. Recent studies have highlighted the crucial roles of non-coding RNAs, derived from the non-protein-coding regions of the genome, in cellular functions and disease progression. These non-coding RNAs (Figure 1) include small nuclear RNAs (snRNA), small nucleolar RNAs (snoRNA), Piwi-interacting RNAs (piRNA), microRNAs (miRNA), long non-coding RNAs (lncRNA), ribosomal RNA (rRNA), and transfer RNA (tRNA) [4]. They participate in the modification and processing of messenger RNAs (mRNA) and regulate a broad range of biological processes. piRNAs, which are small ncRNAs (24–30 nucleotides in length), interact with the PIWI subfamily of the Argonaute proteins and play a pivotal role in preserving genomic stability in germ cells [5]. snoRNAs, ranging from 60 to 300 nucleotides, are integral components of small nucleolar ribonucleoproteins (snoRNPs) and play vital roles in RNA modification and processing [6]. lncRNAs, which are greater than 200 nucleotides in length, represent the largest fraction of the human non-coding transcriptome and are involved in a wide range of regulatory functions [7]. snRNAs, typically 100–300 nucleotides in length, are involved in the formation of small nuclear ribonucleoproteins (snRNPs) and are key players in critical cellular processes such as RNA splicing [8]. miRNAs, approximately 22 nucleotides in length, are key regulators of post-transcriptional gene expression [9]. By binding to complementary sequences on target mRNAs, miRNAs induce mRNA degradation or inhibit translation, thus modulating a variety of biological processes, including cell proliferation, differentiation, and apoptosis [10,11,12]. The dysregulation of miRNAs has been implicated in a wide array of diseases, with a particularly significant role in cancer, where these small but powerful molecules can function either as oncogenes, promoting the growth and proliferation of cancer cells, or as tumor suppressors, inhibiting tumor development and progression. Consequently, miRNAs hold significant potential for cancer therapy [13,14,15].

Among the diverse miRNAs, the miR-200 family, particularly miR-200c, has garnered significant attention in cancer biology due to its role in regulating epithelial–mesenchymal transition (EMT), a critical process in cancer metastasis [16,17,18,19]. The miR-200 family includes five members: miR-141, miR-429, miR-200a, miR-200b, and miR-200c, with miR-200c being the most extensively studied [20]. It is known to target key transcription factors such as *ZEB1* and *ZEB2*, which are pivotal in promoting EMT and enhancing the invasive potential of cancer cells [21,22].

The discovery of miR-200c’s significant involvement in various types of cancers, including but not limited to breast, colorectal, and gastric cancers, underscores its crucial importance in the intricate field of tumor biology, revealing potential pathways for understanding cancer progression and developing targeted therapies. miR-200c has been demonstrated to suppress migration and invasion in triple-negative breast cancer by directly targeting ZEB2, thus inhibiting EMT [16,23]. Additionally, elevated levels of miR-200c have been associated with poor prognosis in patients with cholangiocarcinoma, suggesting its potential as a biomarker for disease progression [24,25].

The purpose of this comprehensive review is to thoroughly explore and elucidate the intricate mechanisms by which miR-200c contributes to the complex processes of tumorigenesis, invasion, and metastasis, while also examining its clinical significance as a promising potential therapeutic target and a valuable biomarker in various types of malignancies that affect patients worldwide. Understanding the intricate role of miR-200c in the complex process of cancer progression could provide valuable insights into the development of novel diagnostic and therapeutic strategies that are specifically aimed at improving patient outcomes and enhancing the overall effectiveness of cancer treatment.

## 2. Biological Characteristics of MicroRNA-200c

### 2.1. Biosynthesis and Regulatory Mechanisms of miR-200c

**Figure 2:**
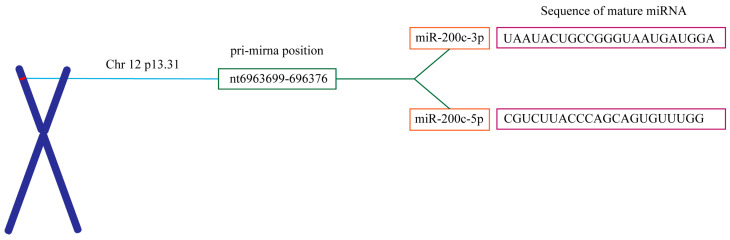
Chromosomal location of miR-200c, position of pre-miRNA hairpins, and sequence of mature miRNA.

miR-200c, a member of the miR-200 family, plays a critical role in regulating key biological processes (Figure 2). Its biosynthesis begins with the transcription of the primary miRNA (pri-miRNA), which is subsequently processed in the nucleus by the Drosha-DGCR8 complex into precursor miRNA (pre-miRNA). This pre-miRNA is then exported to the cytoplasm via exportin-5, where Dicer further processes it to generate the mature miRNA. The expression of miR-200c is tightly regulated by various transcription factors and epigenetic modifications, including DNA methylation and histone modifications [26]. Changes in the expression levels of miR-200c can influence the proliferative and invasive capabilities of tumor cells [27]. Additionally, environmental factors and cellular stress can also influence miR-200c levels, highlighting its role as a responsive biomarker in various pathological conditions, including cancer and chronic inflammation [28].

### 2.2. Target Genes and Functions of miR-200c

MiR-200c has been recognized as a significant regulator that targets multiple genes involved in crucial signaling pathways, which are integral to the complex processes underlying cancer progression and metastasis. One significant target is the phosphatase and tensin homolog (*PTEN*), a well-known tumor suppressor gene. The downregulation of PTEN by miR-200c has been implicated in promoting cell proliferation and invasion in various cancers, including papillary thyroid carcinoma, gastric cancer, and pituitary adenoma [29,30]. In addition to *PTEN*, miR-200c targets vascular endothelial growth factor receptor (*VEGFR*) and matrix metalloproteinase 9 (*MMP9*), both of which play essential roles in angiogenesis and extracellular matrix remodeling, respectively. The inhibition of these targets by miR-200c contributes to reduced migratory and invasive capabilities of cancer cells [27]. Furthermore, miR-200c is also involved in regulating the EMT, a process critical for cancer metastasis. By targeting *ZEB1* and *ZEB2*, miR-200c promotes the maintenance of epithelial characteristics in cells, thereby acting as a tumor suppressor [31]. The multifunctional role of miR-200c in targeting a diverse array of genes highlights its significant potential as both a therapeutic target and a biomarker in the realms of cancer treatment and diagnosis, suggesting that its intricate interactions within cellular pathways could pave the way for innovative strategies to combat this complex disease.

## 3. The Relationship Between MicroRNA-200c and Oncogenesis

MiR-200c is known for its significant role in regulating cancer progression. Its expression levels have been shown to vary across different types of tumors, influencing various cellular processes, including proliferation, apoptosis, pyroptosis, and metastasis. This section will delve into the expression patterns of miR-200c in different tumor types and its implications in tumor biology.

### 3.1. Expression Patterns of miR-200c in Different Tumor Types

The expression of miR-200c is notably dysregulated in various malignancies, including breast, ovarian, and colorectal cancers, where its altered levels can significantly impact tumor progression and metastasis, highlighting its potential role as a crucial biomarker and therapeutic target in the complex landscape of cancer biology [32]. Table 1 illustrates the target genes of miR-200c and the regulatory functions in different tumor types. In triple-negative breast cancer, for instance, miR-200c functions as a tumor suppressor by targeting *ZEB2*, thereby inhibiting EMT and metastasis [16]. Similarly, in epithelial ovarian cancer, miR-200c has been recognized as a master regulator of oncogenes and tumor suppressors, with its expression inversely correlated with the levels of *PPP3CC*, a protein involved in apoptosis [33]. Furthermore, in colorectal cancer, miR-200c-3p has been found to negatively regulate migration and invasion in response to lipopolysaccharide stimulation, indicating its role in tumor progression [34]. The diverse expression patterns of miR-200c across various types of cancers indicate its promising potential to serve not only as a diagnostic biomarker but also as a therapeutic target, highlighting the critical need for further in-depth research to elucidate its precise role and mechanisms in tumor biology, which could ultimately lead to more effective treatment strategies and improved patient outcomes.

### 3.2. The Role of miR-200c in Tumor Cell Proliferation and Apoptosis

miR-200c plays a crucial role in regulating tumor cell proliferation and apoptosis across various cancer types. In Wilms tumor, miR-200c overexpression has been shown to markedly suppress cell proliferation and enhance apoptosis, primarily through the modulation of the Akt signaling pathway [59]. This finding aligns with other studies showing that miR-200c can induce apoptosis and inhibit proliferation in papillary thyroid cancer by directly targeting *DACH1*, a known oncogene [36]. In breast cancer, the loss of the miR-200c/141 cluster promotes the generation of EMT-like breast cancer stem cells, thereby increasing tumor metastasis. In contrast, the direct upregulation of the target genes homeodomain-interacting protein kinase 1 (*HIPK1*) and the activation of β-catenin inhibit tumor growth [37]. Additionally, miR-200c suppresses breast cancer cell growth by increasing intracellular cAMP levels through the inhibition of *PDE7B* expression, leading to cell cycle arrest and apoptosis [38]. Furthermore, miR-200c targets the X-linked inhibitor of apoptosis protein (XIAP), thereby inhibiting the proliferation of triple-negative breast cancer cells and promoting apoptosis [39]. Additionally, in non-small cell lung cancer, miR-200c has been shown to enhance cisplatin sensitivity and suppress malignant behaviors by targeting *RRM2*, a gene associated with drug resistance [40]. In cervical cancer, miR-200c inhibits tumor invasion, migration, and proliferation by targeting *MAP4K4* [41]. In bladder cancer, it directly interacts with the 3′-UTR regions of BMI-1 and E2F3, leading to their downregulation and resulting in decreased invasion, migration, and proliferation of cancer cells [42]. Collectively, these findings emphasize the critical role of miR-200c in tumorigenesis and suggest its potential as a therapeutic target in cancer treatment.

The role of miR-200c in regulating cellular pyroptosis has primarily been investigated in non-cancerous diseases. miR-200c has been shown to target NIMA-related kinase 7 (*NEK7*) to inhibit the activation of the NOD-like receptor 3 (*NLRP3*) inflammasome, thereby reducing cellular inflammation and suppressing pyroptosis in mouse epithelial cells, which in turn improves experimental models of inflammatory bowel disease [60]. However, in the context of diabetes, miR-200c promotes hyperglycemia-induced pyroptosis in human retinal microvascular endothelial cells by targeting *SLC30A7*, thereby contributing to the progression of retinal pathologies. Additionally, miR-200c regulates the expression of *NRF2* to promote pyroptosis in mouse podocyte cell lines, which exacerbates the development of diabetic foot [61,62].

## 4. The Role of MicroRNA-200c in the Invasion Process

### 4.1. Regulation of EMT by miR-200c

EMT is a process in which epithelial cells acquire mesenchymal characteristics, including increased motility and invasiveness, accompanied by the loss of intercellular adhesion [63]. MiR-200c serves as a critical regulator of EMT, a process that is pivotal in cancer metastasis [64]. The downregulation of miR-200c is frequently linked to the promotion of EMT, a crucial biological process that facilitates the transformation of stationary epithelial cells into more mobile mesenchymal cells, ultimately leading to heightened invasiveness and metastasis in a variety of cancers, including but not limited to breast and gastric cancers, where this molecular alteration significantly contributes to the aggressive nature of the disease and the challenges associated with effective treatment [16]. Studies have demonstrated that miR-200c regulates key transcription factors, including ZEB1 and ZEB2, which downregulate epithelial markers such as E-cadherin and upregulate mesenchymal markers like N-cadherin and vimentin [65,66]. This regulatory axis facilitates the transition from an epithelial to a mesenchymal phenotype, enhancing the migratory and invasive capabilities of cancer cells [43,67]. Furthermore, the restoration of miR-200c expression has been shown to reverse EMT characteristics, thereby inhibiting cancer progression and suggesting its potential as a therapeutic target [44,45]. In colorectal cancer, for instance, miR-200c has been implicated in regulating the expression of lncRNA ZFAS1, which in turn influences the *ZEB1*/E-cadherin signaling pathway, emphasizing the complex interplay between miR-200c and other regulatory molecules during EMT [35,68]. Additionally, the upregulation of miR-200c has been associated with a decrease in stemness and invasive traits in breast cancer cells, highlighting its role as a tumor suppressor [69]. Overall, the modulation of miR-200c presents a promising avenue for therapeutic intervention in various cancers characterized by aggressive EMT and metastasis.

### 4.2. Relationship Between miR-200c and Cell Migration and Invasion Capabilities

The relationship between miR-200c and the migratory and invasive abilities of cancer cells is well-established, with numerous studies demonstrating that decreased levels of miR-200c correlate with enhanced cell motility and invasive potential [64]. For example, in breast cancer, the restoration of miR-200c expression has been shown to significantly inhibit cell migration and invasion by targeting and downregulating *ZEB2*, a key player in promoting EMT [16,43,70]. Additionally, miR-200c affects the EMT mechanism by targeting actin-regulatory proteins like *FHOD1*. This interference disrupts the translocation of the serum response factor (*SRF*) coactivator myocardin-related transcription factor A, leading to reduced *SRF* expression and transcriptional activity. Ultimately, this results in the downregulation of *SRF* target genes, including myosin light chain 2 (*MLC2*), affecting stress fiber formation and contraction [46]. This effect is further supported by findings that miR-200c can modulate the expression of integrins, which are crucial for cell adhesion and migration processes [71].

In NSCLC, miR-200c suppresses EMT, invasion, and migration by targeting and downregulating HMGB1 expression [47]. Moreover, the overexpression of miR-200c-3p has been linked to increased sensitivity to chemotherapy, suggesting that miR-200c not only inhibits invasion but also enhances the efficacy of cancer treatments by modulating cell behavior [40]. Lactate dehydrogenase A (*LDHA*) is also a target of miR-200c, which suppresses the proliferation and migration of NSCLC by downregulating *LDHA* expression [48]. In gastric cancer, miR-200c specifically binds to the 3′-UTR of *FN1* and *KLF6*, thereby downregulating their expression and affecting the proliferation, migration, and invasion of gastric cancer cells [49,50]. In pancreatic cancer, miR-200c also influences migration and invasion by targeting the mRNA of *MUC4* and *MUC16* [51]. These significant findings collectively highlight the dual role of miR-200c, which not only suppresses invasive characteristics in cancer cells but also enhances their responsiveness to treatment, thereby making it a critical focus for researchers aiming to develop effective therapeutic strategies against metastatic cancers [72,73].

## 5. MicroRNA-200c and the Mechanisms of Tumor Metastasis

### 5.1. The Role of miR-200c in the Expression of Metastasis-Related Genes

MiR-200c is a critical regulator in the metastatic progression of various cancers, particularly breast cancer, by modulating *ZEB1* and *ZEB2*, which in turn influence the EMT process. Studies have demonstrated that the downregulation of miR-200c correlates with increased levels of these factors, leading to enhanced tumor cell motility and invasiveness [73]. miR-200c inhibits tumor metastasis by downregulating the expression of the transcription factor *c-Jun* and *MRTF*/*SRF*, which in turn interferes with the expression of the cytoskeletal protein filamin A, thereby reducing the motility of tumor cells [25]. Furthermore, the loss of miR-200c can give rise to a more aggressive cancer stem cell phenotype, which is associated with increased metastatic potential. Deletion of the miR-200c/141 cluster in breast cancer models has been associated with the induction of EMT-like traits in cancer stem cells, leading to enhanced tumor metastasis [37]. In head and neck squamous cell carcinoma (HNSCC), miR-200c suppresses HNSCC -associated cancer stem cells by targeting the 3′ UTR of *BMI1*, thereby reducing the tumor’s metastatic potential [52]. This compelling evidence underscores the essential and pivotal role of miR-200c as a crucial tumor suppressor, highlighting the fact that its expression is inversely associated with the aggressive metastatic behavior of various cancers. This relationship suggests that higher levels of miR-200c may inhibit the spread of cancer cells, making it a promising and potential therapeutic target for innovative strategies aimed at preventing tumor progression and the subsequent metastasis.

### 5.2. The Interaction of miR-200c with the Tumor Microenvironment

The tumor microenvironment significantly influences cancer progression and metastasis, and miR-200c plays a pivotal role in this dynamic [74]. Recent research has shown that miR-200c can modulate the behavior of tumor-associated macrophages, which are crucial components of the tumor microenvironment. Specifically, miR-200c has been found to be transferred from apoptotic tumor cells to macrophages, where it can downregulate migration-associated genes, thereby reducing macrophage infiltration into tumor sites [75]. In triple-negative breast cancer, miR-200c has been shown to promote tumor progression by upregulating *PAI-2*, thereby regulating the polarization of M2 phenotype macrophages [76]. miR-200c expression has also been correlated with the density of tumor-infiltrating T cells in colorectal cancer, with high levels of miR-200c associated with poor overall survival [77]. Furthermore, the miR-200 family has a close relationship with the expression of the immune checkpoint protein programmed cell death ligand 1 (*PD-L1*) [78,79,80,81]. In non-small cell lung cancer, 6-gingerol has been found to induce miR-200c expression while downregulating *PD-L1* expression [53]. Chen et al. reported that the seed sequences of miR-200 (miR-200a and miR-200b/c) can bind to the 3′-UTR of *PD-L1*, leading to its downregulation [54]. In contrast, the downregulation of miR-200 results in increased *PD-L1* expression, which suppresses CD8+ T cell activity in the tumor microenvironment and promotes metastasis. In hepatitis B virus (HBV)-induced hepatocellular carcinoma, the oncofetal antigen *SALL4* upregulates *PD-L1* expression by inhibiting the transcription of miR-200c, leading to the exhaustion of antiviral CD8+ T cells [55]. Additionally, doxorubicin and miR-200c dual-vector nanoparticle drugs effectively inhibit *PD-L1* expression and induce immunogenic cell death, potentially enhancing the efficacy of cancer therapy [82]. In bladder cancer, miR-200a-3p from the miR-200 family has also been shown to bind to the 3′-UTR of *PD-L1* [83].

This interaction suggests that miR-200c not only influences tumor cell behavior but also modifies the immune landscape of the tumor, potentially leading to a less favorable environment for tumor growth and spread. Additionally, miR-200c has been implicated in the regulation of the exosomal cargo of cancer cells, with studies indicating that it can be sorted into exosomes, which then influence the behavior of distant cells and contribute to the metastatic process [84]. This dual role of miR-200c in both tumor cell regulation and interaction with the tumor microenvironment highlights its complexity and importance as a target for therapeutic intervention in metastatic cancers. By targeting the pathways associated with miR-200c, it may be possible to alter the tumor microenvironment and inhibit metastasis effectively.

## 6. Clinical Prospects of MicroRNA-200c

MiR-200c has emerged as a significant player in various cancer types, demonstrating its potential as a biomarker and therapeutic target. Its expression is often dysregulated in malignancies, and understanding its clinical implications can pave the way for innovative diagnostic and therapeutic strategies.

### 6.1. Potential of miR-200c as a Biomarker

The potential of miR-200c as a biomarker has been widely investigated in various cancers. In oral squamous cell carcinoma, for example, reduced miR-200c levels correlate with advanced disease stages and poor prognosis, indicating that it could serve as an independent predictor for recurrence-free survival and overall survival [85]. Similarly, in papillary thyroid cancer, high expression levels of miR-200c correlated with poor clinical outcomes, suggesting that it may play a pivotal role in tumor progression by modulating pathways such as the *PTEN* signaling cascade [29]. In breast cancer, miR-200c demonstrates high accuracy in both diagnosis and prognostic assessment [86]. A systematic review has indicated that low expression of miR-200c in tumor tissues of colorectal cancer is associated with poorer survival prognosis, while elevated levels of miR-200c in blood are linked to worse clinical outcomes [17]. Moreover, research has shown that miR-200c levels in the serum and tissues of Crohn’s disease patients reflect disease activity, further supporting its utility as a biomarker beyond oncology [87]. The diagnostic capabilities of miR-200c extend to chronic periodontitis, where its expression in gingival crevicular fluid demonstrated a strong correlation with clinical parameters, reinforcing its potential as an early diagnostic tool [20]. These significant findings collectively underscore the remarkable versatility of miR-200c as a biomarker across various pathologies, thereby enhancing its appeal and potential utility for clinical applications in the medical field, where it could serve as a crucial tool for diagnosis, prognosis, and personalized treatment strategies.

### 6.2. Targeted Therapeutic Strategies Based on miR-200c

miR-200c has been implicated in the chemosensitivity of various cancers [64,88]. In gastric cancer, it targets the 3′ UTRs of ZEB2 and RhoE, leading to their downregulation and enhancing sensitivity to cisplatin-based chemotherapy [56,89]. Additionally, in breast cancer, miR-200c may enhance sensitivity to microtubule-targeting chemotherapy drugs through class III β-tubulin (*TUBB3*) [57]. miR-200c also plays a critical role in the development of resistance to 5-fluorouracil in ovarian cancer. lncRNA TMPO-AS1 promotes ovarian cancer cell metastasis and chemoresistance by suppressing miR-200c and upregulating *TMEFF2*, which activates the PI3K/Akt signaling pathway [58]. Additionally, miR-200c is involved in resistance to the targeted therapy trastuzumab. TGF-β downregulates miR-200c expression, leading to the upregulation of the transcription factors *ZEB1* and *ZEB2*. This, in turn, induces trastuzumab resistance and enhances the invasiveness and migratory capacity of cancer cells [90,91].

The therapeutic potential of miR-200c has gained significant traction in recent years, particularly in the context of targeted therapies, as researchers and clinicians alike are increasingly recognizing its promising role in enhancing treatment efficacy and improving patient outcomes in various diseases [89]. In breast cancer, for example, the delivery of miR-200c-3p using tumor-targeted nanoparticles has shown promise in inhibiting tumor growth and metastasis by downregulating oncogenes like *ZEB1* and *ZEB2* [73]. This targeted delivery system, which has been meticulously designed and developed, not only enhances the therapeutic efficacy of the treatment by ensuring that the medication is delivered directly to the cancerous cells, thereby maximizing its effectiveness, but it also minimizes systemic toxicity, which is often a significant concern in traditional cancer therapies, representing a remarkable and significant advancement in the evolving landscape of cancer treatment strategies that could potentially improve patient outcomes and quality of life. Furthermore, in glioblastoma, the overexpression of miR-200c has been proposed as a strategy to overcome chemotherapy resistance, as it was found to downregulate metabolic enzymes involved in glycolysis, thus impairing tumor cell metabolism and enhancing sensitivity to standard treatments like temozolomide [92]. Additionally, innovative approaches combining miR-200c with other therapeutic agents, such as *PD-L1* inhibitors and BRAF-targeted therapies, have demonstrated improved anti-tumor responses by enhancing immune cell infiltration into tumors [93]. These significant findings highlight the multifaceted role of miR-200c in both diagnostic and therapeutic contexts, suggesting that it may play a crucial part in the development of personalized medicine strategies tailored specifically for cancer patients.

## 7. Summary

In conclusion, miR-200c emerges as a multifaceted player in the processes of tumorigenesis, invasion, and metastasis. Its involvement in these critical pathways underscores its importance in cancer biology, as it regulates various cellular functions through the modulation of target genes. The dual role of miR-200c—acting both as a tumor suppressor and an oncogene in different contexts—highlights the complexity of its function in cancer progression and necessitates a nuanced understanding of its mechanisms of action (Table 2).

As we evaluate the potential of miR-200c as a biomarker and therapeutic target, it is essential to acknowledge the current discrepancies in research findings. Various studies have reported contrasting effects of miR-200c on tumor behavior, which can be attributed to factors such as tumor type, microenvironment, and even the specific genetic background of the tumor cells. Balancing these differing viewpoints requires a comprehensive approach that considers the context-dependent nature of miR-200c’s role in cancer.

In order to significantly enhance and deepen our understanding of the multifaceted roles of miR-200c, it is imperative that future research endeavors concentrate on meticulously unraveling the intricate molecular mechanisms by which this microRNA exerts its diverse effects across a wide array of cancer types. This exploration will not only shed light on the fundamental biological processes involved but also pave the way for potential therapeutic applications and interventions that may revolutionize cancer treatment strategies. This comprehensive exploration encompasses a meticulous investigation into the intricate pathways that this entity influences, a thorough identification of novel target genes that may play pivotal roles in various biological processes, and a deep understanding of the complex mechanisms that regulate its expression across a spectrum of diverse physiological and pathological conditions, shedding light on the multifaceted interactions at play.

Ultimately, the investigation of miR-200c holds great promise for enhancing our understanding of cancer biology and the development of innovative therapeutic strategies. By harmonizing diverse research findings and fostering collaborative studies, the scientific community can better leverage the potential of miR-200c in clinical applications, providing insights for more effective cancer diagnostics and treatments.

## Figures and Tables

**Table 1:** Target genes of miR-200c and the functions regulated by miR-200c.

Target Gene	Regulatory Function	Cancer Type	Reference
*ZEB1* and *ZEB2*	Migration, invasion, and metastasis	(TNBC) Breast cancer and colorectal cancer	[16,34,35]
*KRAS*	Proliferation	(TNBC) Breast cancer	[23]
*c-Jun*	Migration and invasion	(HR+ and TNBC) Breast cancer	[25]
*VEGFR*	Migration and invasion	Gastric cancer	[27]
*MMP9*	Migration and invasion	Gastric cancer	[27]
*RhoE*	Cisplatin sensitivity	Gastric cancer	[27]
*PTEN*	Proliferation, migration, and invasion	Papillary thyroid cancer, gastric cancer, and pituitary adenoma	[29,30]
*PPP3CC*	Apoptosis	Epithelial ovarian cancer	[33]
*DACH1*	Proliferation	papillary thyroid cancer	[36]
*HIPK1*	Proliferation and metastasis	(HR+ and HER2+ and TNBC) Breast cancer	[37]
*PDE7B*	Proliferation	(TNBC) Breast cancer	[38]
*XIAP*	Proliferation	(TNBC) Breast cancer	[39]
*RRM2*	Proliferation and cisplatin sensitivity	Non-small cell lung cancer	[40]
*MAP4K4*	Proliferation, migration, and invasion	Cervical cancer	[41]
*E2F3*	Proliferation, migration, and invasion	Bladder cancer	[42]
*HIF1-α*	Proliferation, migration, and invasion	(HR+ and TNBC) Breast cancer	[43]
*QKI-5*	Migration and invasion	Renal clear cell cancer	[44]
*EP300*	Proliferation, migration, and invasion	Wilms tumor	[45]
*FHOD1*	Migration and invasion	(HR+ and TNBC) Breast cancer	[46]
*PPM1F*	Migration and invasion	(HR+ and TNBC) Breast cancer	[46]
*HMGB1*	Migration and invasion	Non-small cell lung cancer	[47]
*LDHA*	Proliferation, migration, and invasion	Non-small cell lung cancer	[48]
*FN1*	Proliferation, migration, and invasion	Gastric cancer	[49]
*KLF6*	Migration and invasion	Gastric cancer	[50]
*MUC4* and *MUC16*	Metastasis	Pancreatic cancer	[51]
*BMI1*	Proliferation and metastasis	Head and neck squamous cell cancer and bladder cancer	[52]
*PD-L1*	Metastasis	Non-small cell lung cancer	[53,54,55]
*ZEB2*	Cisplatin sensitivity	Gastric cancer	[56]
*TUBB3*	Sensitivity to microtubule-targeting chemotherapeutic agents	Endometrial cancer and ovarian cancer	[57]
*TMEFF2*	Migration, invasion, metastasis, and 5-FU sensitivity	Ovarian cancer	[58]

Abbreviation: TNBC = triple-negative breast cancer; HR = hormone receptor; HER2 = human epidermal growth factor receptor 2.

**Table 2:** The tumor suppressor gene and oncogene roles of microRNA 200c.

Functional Category	Cancer Type	Mechanism	Reference
Tumor suppressor gene	Breast cancer	Downregulates *ZEB1* and *ZEB2*, inhibiting EMT and reducing tumor cell invasiveness.	[16,22]
		Downregulates *CHK1* through the inhibition of long non-coding RNA LINC02582, increasing radiosensitivity.	[21]
		Downregulates *KRAS*, suppressing breast cancer cell proliferation.	[23]
		Downregulates filamin A, a cytoskeletal component, inhibiting breast cancer metastasis.	[25]
		Regulates breast cancer stem cell heterogeneity, suppressing tumor metastasis.	[37]
		Downregulates *PDE7B*, inhibiting tumor cell proliferation.	[38]
		Downregulates *XIAP*, inhibiting tumor cell proliferation and promoting apoptosis.	[39]
		Downregulates *FHOD1* and *PPM1F*, inhibiting migration and invasion of breast cancer cells.	[46]
	Gastric cancer	Downregulates *RhoE*, *VEGFR*, and *MMP9*, increasing cisplatin sensitivity.	[27]
		Downregulates *FN1*, inhibiting tumor cell proliferation, migration, and invasion.	[49]
		Downregulates *ZEB2*, enhancing cisplatin sensitivity.	[56]
	Colorectal cancer	Downregulates *ZEB1* and *ZEB2*, inhibiting EMT and reducing tumor cell invasiveness.	[34,68]
	Wilms tumor cells	Reduces Akt phosphorylation and its downstream protein GLUT1 expression, promoting apoptosis and inhibiting cell proliferation.	[59]
	Bladder cancer	Downregulates *BMI-1* and *E2F3*, inhibiting EMT and reducing tumor cell invasion and proliferation.	[42]
		Inhibits Akt2/mTOR signaling pathway, affecting the expression of *VEGF* and *HIF-1α*, regulating tumor angiogenesis.	[72]
	Non-small cell lung cancer	Downregulates *RRM2*, enhancing cisplatin sensitivity and inhibiting tumor proliferation.	[41]
		Downregulates *HMGB1*, inhibiting EMT, migration, and invasion of lung cancer cells.	[47]
		Downregulates *LDHA*, inhibiting NSCLC cell proliferation and migration.	[61]
	Cervical cancer	Downregulates *MAP4K4*, inhibiting cervical cancer cell proliferation and progression.	[41]
	Clear cell renal carcinoma	Downregulates *QKI-5*, inhibiting EMT and reducing tumor cell invasiveness.	[44]
	Nephroblastoma cells	Downregulates *EP300* and inactivates AKT/FOXO1/p27 pathway to suppress tumor cell proliferation and invasion.	[45]
	Pancreatic cancer	Downregulates *MUC4* and *MUC16*, inhibiting tumor cell invasiveness.	[51]
	Head and neck squamous cell carcinoma	Downregulates *BMI1/ZEB1*, inhibiting EMT and reducing tumor cell invasiveness.	[52]
	HBV-related hepatocellular carcinoma	Downregulates *PD-L1*, reversing antiviral CD8 T cell exhaustion.	[55]
	Ovarian cancer	Downregulates *TMEFF2*, inhibiting EMT, reducing tumor cell proliferation and invasion, and suppressing 5-FU resistance.	[58]
Oncogene	Papillary thyroid cancer	Downregulates *PTEN*, promoting tumor cell proliferation, migration, and invasion.	[29]
	Pituitary adenoma cells	Downregulates *PTEN*, promoting tumor cell proliferation, migration, and invasion.	[30]
	Ovarian cancer	Downregulates *PPP3CC*, inhibiting tumor cell apoptosis.	[33]
	Breast cancer	Promotes *VEGF-A* secretion, activating FAK and PI3K/AKT signaling pathways, thereby enhancing cell migration and invasion.	[70]
		Promotes *PAI-2* secretion and M2 macrophage polarization, facilitating tumor cell metastasis.	[76]
	Gastric cancer	Downregulates *KLF6*, promoting tumor cell proliferation, migration, and invasion.	[50]
